# Evaluation of the Antioxidant, Anti-Inflammatory, and Anticancer Activities of *Euphorbia hirta* Ethanolic Extract

**DOI:** 10.3390/molecules190914567

**Published:** 2014-09-15

**Authors:** Neelesh Sharma, Kalpa W. Samarakoon, Rajendra Gyawali, Yang-Ho Park, Sung-Jin Lee, Sung Jong Oh, Tae-Hoon Lee, Dong Kee Jeong

**Affiliations:** 1 Department of Animal Biotechnology, Faculty of Biotechnology, Jeju National University, Jeju 690-756, Korea; E-Mails: drneelesh_sharma@yahoo.co.in (N.S.); ohsjong@gmail.com (S.J.O.); 2 Department of Marine Life Science, Jeju National University, Jeju 690-756, Korea; E-Mail: kalpa.samarakoon@gmail.com; 3 Faculty of Biotechnology, School of Applied Life Science, Jeju National University, Jeju 690-756, Korea; E-Mail: ragyawali@gmail.com; 4 BRM Institute, Seoul 135-010, Korea; E-Mail: brmlife@brmrnd.com; 5 Department of Animal Biotechnology, School of Animal Life Sciences, Kangwon National University, Chuncheon 200-701, Korea; E-Mail: sjlee@kangwon.ac.kr; 6 Department of Oral Biochemistry, Dental Science Research Institute, Chonnam National University, Gwangju 500-757, Korea; E-Mail: thlee83@chonnam.ac.kr

**Keywords:** *Euphorbia hirta*, chemical composition, online HPLC-ABTS^+•^ bioassay, antioxidant, anti-inflammatory, anticancer

## Abstract

This study evaluated the chemical composition, antioxidant, anti-inflammatory and anticancer activities of a *Euphorbia hirta* L. extract. The antioxidant activities of whole *E. hirta* ethanol extract were determined by electron spin resonance spectrophotometric analysis of 1,1-diphenyl-2-picryl-hydrazyl (DPPH), hydroxyl, and alkyl radical levels and by using an online high-performance liquid chromatography (HPLC)-2,2'-azino-bis(3-ethylbenzothiazoline-6-sulfonic acid) assay. The *E. hirta* ethanol extract (0.5 mg/mL) exhibited DPPH-scavenging activity of 61.19% ± 0.22%, while the positive control (0.5 mg/mL ascorbic acid) had 100% ± 0.22% activity. The concentration of the extract required to trap 50% of DPPH (IC_50_) was 0.205 mg/mL. Online HPLC analysis of the extract also showed strong antioxidant activity. The anti-inflammatory activity of the *E. hirta* extract was assessed in lipopolysaccharide-induced RAW 264.7 macrophages. The anti-inflammatory activity was highest in the presence of 200 µg/mL *E. hirta* extract, and nitric oxide production was decreased significantly (*p <* 0.05). The extract also showed selective anticancer activity at a concentration of 100 µg/mL (*p* < 0.05). These results indicated that *E. hirta* may warrant further investigation for the development of antioxidant, anti-inflammatory, and anticancer herbal medications.

## 1. Introduction

Constituents of several medicinal plants have been used since ancient times to treat a variety of diseases. Approximately 70,000 plant species have been used for medicinal purposes. The total global market for herbal remedies (excluding soy, algae, and fiber) is currently worth approximately $83 billion [1], which highlights the magnitude of this industry. In many developing countries, one-third of the population relies on traditional practitioners and medicinal plants to meet its primary health care needs [2]. According to the World Health Organization, an estimated 80% of people worldwide are interested in traditional medicine. At present, research evaluating the chemical compositions and therapeutic utilities of herbal medicines is being conducted worldwide.

Reactive oxygen species (ROS) include a variety of free radicals such as superoxide anion (O_2_**˙**^−^), hydroxyl radical (OH**˙**), nitric oxide radical (NO**˙**), and peroxyl radical (RO_2_**˙**^−^), and non-free radical species such as hydrogen peroxide (H_2_O_2_) [3]. Normal cells have a natural ROS defense system that effectively eliminates radicals through enzyme-mediated systems such as superoxide dismutase, peroxidase, catalase, and glutathione peroxidase; however, excessive production of ROS causes cellular damage and results in oxidative stress. Nitric oxide (NO) is synthesized by many cell types involved in immunity and inflammation. It is a signaling molecule that plays a key role in the pathogenesis of inflammation [4].

*Euphorbia hirta* L. belongs to the Euphorbiaceae family and is a very popular herb amongst practitioners of traditional herbal medicine. There are approximately 1600 species in the Euphorbia genus, and many of these are grown throughout Asia because of their pharmacological properties. Traditional medicine practitioners generally use aqueous extracts of various *E. hirta* parts. These extracts have been used to treat a variety of diseases, including cough, hay asthma, bowel disease, worm infestation, kidney stones, and bronchial disease, as well as decrease lactation; they have also been used for their sedative, anxiolytic, analgesic, antipyretic, and anti-inflammatory properties [4,5]. The whole *E. hirta* plant has been reported to show 45% immunomodulation activity by inhibiting NO production [6]. Previous studies have attempted to characterize the chemical compounds present in *E. hirta* [7,8] but there is a lack of information relating to the specific constituents of *E. hirta* and their potential clinical applications. The aim of the present study was to investigate the *in vitro* antioxidant activity of *E. hirta* and to evaluate its potential for clinical use as a natural antioxidant, anti-inflammatory, and anticancer agent.

## 2. Results and Discussion

### 2.1. Gas Chromatography-Mass Spectrometry (GC-MS) of Ethanolic Extract of E. hirta

**Figure 1:**
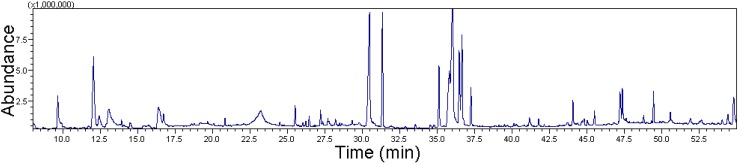
GC-MS chromatogram of ethanol extract of whole plant of *E. hirta* showing the different peaks of compounds present in the extract.

Ethanol extraction of whole *E. hirta* specimens yielded an extract corresponding to 9.65% (w/w) of the initial material. To identify the various constituents of the extract, it was subjected to GC-MS analysis (Figure 1). The characteristics, including the retention time (RT), molecular formula, molecular weight (MW), and relative peak area percentage (peak area relative to the total peak area), of the *E. hirta* extract are summarized in Table 1.

**Table 1:** Chemical composition of ethanolic extract of *E. hirta* tentatively identified by GC-MS.

S. No.	RT ^a^	Name of Compounds ^b^	Molecular Formula	MW	Area (%) ^c^
1	9.66	2,3-Dihydro-3,5-dihydroxy-6-methyl-4*H*-pyran-4-one	C_6_H_8_O_4_	144	2.54
2	12.06	5-Hydroxymethyl-2-furancarboxaldehyde	C_6_H_6_O_3_	126	7.82
3	16.39	1,2,3-Trihydroxybenzene	C_6_H_6_O_3_	126	3.72
4	25.51	Myristic acid	C_14_H_28_O_2_	228	1.03
5	30.48	Pentadecylic acid	C_15_H_30_O_2_	242	13.27
6	31.34	Ethyl palmitate	C_18_H_36_O_2_	284	7.47
7	35.11	Phytol	C_20_H_40_O	296	3.93
8	35.81	Methyl linoleate	C_19_H_34_O_2_	294	7.29
9	36.00	9,12,15-Octadecatrien-1-ol	C_18_H_32_O	264	18.56
10	36.47	7,10-Octadecadienoic acid methyl ester	C_19_H_34_O_2_	294	6.22
11	36.65	Ethyl linoleolate	C_20_H_34_O_2_	306	7.7
12	37.25	Ethyl stearate	C_20_H_40_O_2_	312	2.18
13	48.78	Ethyl hexadecanoate	C_18_H_36_O_2_	284	0.44
14	49.43	Squalene	C_30_H_50_	410	1.63
15	54.41	gamma-Tocopherol	C_28_H_48_O_2_	417	0.76
					84.56 **^#^**

^a^ Retention Time on compounds on Rtx-5MS capillary column. ^b^ Compounds tentatively identified based on retention time and elution order as well as the fragmentation pattern described in the literature. ^c^ Relative peak area percentage (peak area relative to the total peak area %). ^#^ Sum of peak area percentage in the GC-MS chromatogram.

The total peak area was 84.56% in the analysis plot. GC-MS analysis revealed 15 unique compounds in the *E. hirta* ethanol extract. The prevailing compounds were 9,12,15-octadecatrien-1-ol and pentadecylic acid, comprising 18.56% and 13.27% relative peak areas of the 84.56% total peak area, respectively.

The compound 2,3-dihydro-3,5-dihydroxy-6-methyl-4*H*-pyran-4-one (DDMP) has been reported as a major contributor to the anticancer effects of *E. hirta* [9]. In the present study, GC-MS analysis showed that DDMP occupied a 2.54% relative area.

Pharmacological studies of hydroxymethyl-2-furancarboxaldehyde (5-HMF) showed that it had anti-inflammatory activity [10,11,12] and protected against carbon tetrachloride (CCl_4_)-induced injury to the liver and vascular endothelium [13].

Fatty acids regulate a variety of enzymatic processes and may control several chronic inflammatory diseases. The antibacterial and anti-inflammatory properties of nonacetylated fatty acids such as myristic acid (MA) are well-characterized. Phytol is a branched long-chain aliphatic alcohol that has various biological effects, such as peroxisome proliferation in skin sebaceous glands and anti-inflammatory effects in the liver [14]. In the present study, we found that 9,12,15-octadecatrien-1-ol had an 18.56% relative peak area on GC-MS analysis. In a previous study, 9,12,15-octadecatrien-1-ol was shown to have antioxidant and antibacterial activities against *Staphylococcus aureus* ATCC 6538 [15]. Based on the known biological properties of these compounds identified in the *E. hirta* extract, the extract may possess antioxidant, anti-inflammatory, and anticancer activities.

### 2.2. Antioxidant Activity

**Figure 2:**
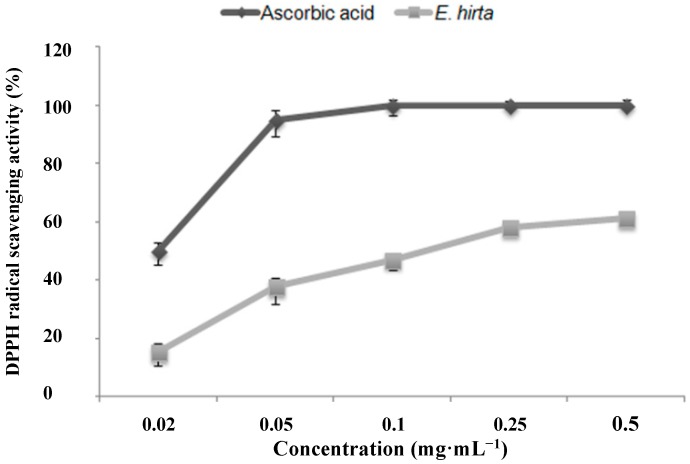
DPPH radical scavenging activity of the ethanol extract of *E. hirta*. Values are the average of triplicate experiments and represented as mean ± SDs.

The *E. hirta* extract showed significant antioxidant activity under several different conditions. Free radical scavenging potential was assessed against 1,1-diphenyl-2-picrylhydrazyl (DPPH) radical. The results of the DPPH assay showed 61.19% ± 0.22% antioxidant activity in the presence of 0.5 mg/mL *E. hirta* ethanol extract (Figure 2). *E. hirta* showed dose-dependent reducing power when compared to those of a standard, ascorbic acid. Antioxidant capacity was expressed as the IC_50_, which was defined as the concentration of antioxidant needed to trap 50% of DPPH. The IC_50_ of the *E. hirta* extract was 0.205 ± 0.21 mg/mL, while that of ascorbic acid, a well-known antioxidant, was 0.020.14 ± 0.04 mg/mL. 

**Figure 3:**
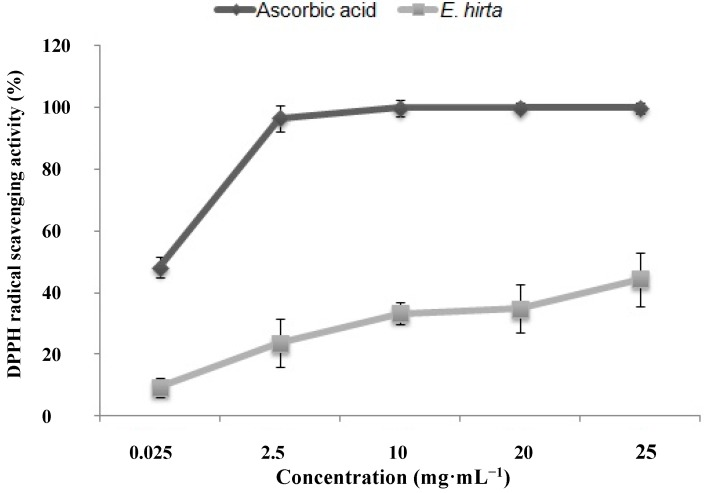
Hydroxyl radical scavenging activity of the ethanol extract of *E. hirta*. Values are the average of triplicate experiments and represented as mean ± SDs.

OH**˙** is a highly reactive species, and attacks nearly all biological molecules through the Fenton reaction, catalyzed by metal ions (Cu^2+^ or Fe^2+^) [16]. The OH**˙-**scavenging activity of the *E. hirta* extract was measured as the percent inhibition of OH**˙** generation by the Fenton reaction, determined using electron spin resonance (ESR) spectrophotometry. We observed that in scavenging the OH**˙** radicals, the extract was not as efficacious as it was against DPPH radicals. The highest antioxidant activity in scavenging OH**˙** radicals was 44.37% ± 8.79%, observed in the presence of 25 mg/mL *E. hirta* extract (Figure 3). Phenolic compounds are also considered important constituents owing to their ability to scavenge hydroxyl groups [17]. OH**˙** can remove hydrogen atoms from cell membranes and induce lipid peroxidation [18]. The antioxidant activities of phenolic compounds are attributed mainly to their redox properties, and these compounds can play an important role in absorbing and neutralizing free radicals and triplet oxygen, and in quenching singlet or decomposing peroxides [19].

In this study, the *E. hirta* extract was able to scavenge DPPH, OH**˙**, and 2,2'-azino-bis (3-ethylbenzothiazoline-6-sulfonic acid) (ABTS^+•^) free radicals. It has been reported that two hydroxyls bonded in the *meta* position, as found in 1,2,3-trihydroxybenzene (present in *E. hirta*), showed higher antioxidant and lower DPPH- and H_2_O_2_-scavenging activity [20]. Antioxidant activity of *E. hirta* was also reported in a Malaysian study that evaluated a methanolic *E. hirta* extract [21].

*E. hirta* ethanol extract contains various compounds with antioxidant properties such as squalene, 9,12,15-octadecatrien-1-ol, phenolic compounds, and others. Prior *in vitro* experiments indicated that squalene was a highly effective oxygen-scavenging agent. Moreover, squalene has shown protective activities against several carcinogens [22]. Phenolic acids with two hydroxyl groups attached to an aromatic ring in the *ortho* position were also reported to have strong antioxidant activity.

Numerous studies have described a range of methods for direct detection of radical initiators and intermediates involved in lipid peroxidation, but the high reactivity of these radical species can prevent them from reaching the steady-state levels required for direct detection. For instance, HO**˙** reacts with most organic molecules at diffusion-controlled rates [23]. The low sensitivity of endogenous radical detection in biological systems can be addressed by spin trapping [24], which is the most direct method used to detect highly reactive free radicals. In the present study, we employed the ESR technique to assess the antioxidant potential of *E. hirta*.

### 2.3. On-Line HPLC-ABTS^+•^ Radical Scavenging Analysis

**Figure 4:**
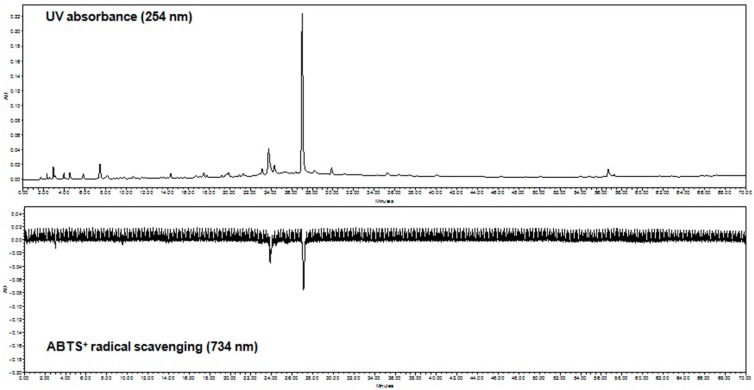
On-line HPLC-ABTS^+•^ radical quenching 2D chromatograms of *E. hirta* crude ethanol extract. A 10 μL of *E. hirta* extract was analyzed by gradient reverse-phase-HPLC with a DAD at 254 nm (positive trace) prior to reaction with ABTS^+•^ radical and the analysis of antioxidant potential at 734 nm (negative trace).

An on-line HPLC-ABTS^+•^ method was used to rapidly identify the radical scavenging activity of the *E. hirta* ethanol extract. As shown in Figure 4, the chromatogram showed high peaks indicative of high radical scavenging activity of this ethanol extract, which was also shown photometrically as a negative peak at 734 nm. The current results showed two peaks with retention times at 23.50 and 27.25 min, suggesting potential free radical scavenging activity of *E. hirta*.

### 2.4. Effects of E. hirta on Lipopolysaccharide (LPS)-Induced NO Production

**Figure 5:**
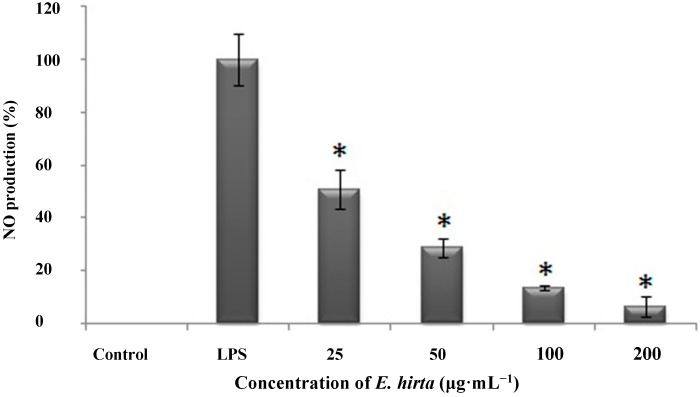
Effect of ethanol extract of *E. hirta* on LPS-induced NO levels in mouse macrophages (RAW 264.7 cells). Cells (1 × 10^5^) were incubated with *E. hirta* (0, 25, 50, 100 and 200 μg/mL) for 1 h, then with LPS (1 μg/mL) for 24 h. Culture supernatants were subsequently isolated and analyzed for nitrite levels. Data represent the means ± SDs of three independent experiments. * *p* < 0.05 *vs.* the non-treated LPS induced group.

NO production in tissues is an indicator of inflammation under several conditions. To investigate the anti-inflammatory properties of *E. hirta*, we used RAW 264.7 cells, which produce NO when stimulated by LPS. The cells were pre-incubated with varying concentrations of *E. hirta* extract (25, 50, 100 and 200 μg/mL) for 1 h, and then stimulated with 1 μg/mL LPS for 24 h. We used two control groups; the first was not exposed to LPS or *E. hirta*, and the second control group was incubated in LPS alone without *E. hirta* pretreatment (LPS treated cells). When the nitrite levels in the media were determined, we observed that *E. hirta* significantly reduced NO production in a dose-dependent manner (Figure 5), as compared to the non-treated LPS-induced cells. This remarkable NO inhibition suggested that *E. hirta* had anti-inflammatory effects.

The anti-inflammatory activity of *E. hirta* extract may be due to the presence of various compounds such as phytol [14], fatty acids [25], 5-HMF [10,11,12], and others. *E. hirta* also contains various other compounds with anti-inflammatory potential such as glucosides, tannins, and flavones, that have been shown to inhibit NO production in earlier reports [7]. In this study, several anti-inflammatory compounds such as 5-HMF, MA, and others were identified as constituents of the *E. hirta* extract and these may be responsible for its anti-inflammatory activity.

NO is generated by macrophages during antigen presentation to T cells [26]. The activated T helper (Th) cells express co-stimulatory molecules that together with several cytokines including IFN-γ, induce NO production in the macrophage. *E. hirta* extract produced strong anti-inflammatory effects by significantly decreasing NO production. The inflammatory reaction is characterized by the production of prostaglandins, cytokines, and inducible NO synthase (iNOS), which generates NO [27]. Uncontrolled NO production can result in nitrosative stress, which may damage protein and DNA, leading to cell injury and death [28,29]. Excessive levels of inflammatory cytokines stimulate NO production in the tissues [30]. NO is responsible for vasodilatation, increased vascular permeability, and edema [31]. The mechanism of NO inhibition may involve prevention of iNOS activity and could contribute to the suppression of PG generation [32].

Several studies of 5-HMF have confirmed its anti-inflammatory activity [10,11,12] and in recent years, it has been used more frequently in traditional Chinese medicine [33,34,35,36]. *E. hirta* also contains 5-HMF, which suggests that it has the potential to act as an anti-inflammatory agent.

### 2.5. Cytotoxicity Assessment of E. hirta Extract on Cell Lines in Vitro

**Figure 6:**
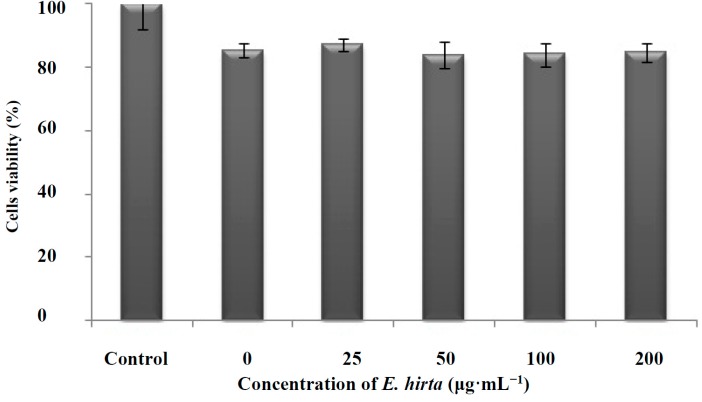
Effect of ethanol extract of *E. hirta* on the viability of LPS-stimulated mouse macrophages (RAW 264.7 cells). Cells (1 × 10^5^) were incubated with *E. hirta* (0, 25, 50, 100, 200 μg/mL) at 37 °C for 24 h. Cells viabilities were evaluated using MTT assays. Data represent the mean ± SDs of three independent experiments.

After evaluating the antioxidant potential and anti-inflammatory properties of *E. hirta*, we assessed the cytotoxic effect of this plant extract in cell culture. We examined the effects of *E. hirta* extract on the viability of RAW 264.7 cells *in vitro* by incubating the cells in 0 (ethanol only), 25, 50, 100, or 200 μg/mL *E. hirta* extract for 24 h; the cell viability was then measured by 3-(4,5-dimethylthiazol-2-yl)-2,5-diphenyltetrazolium bromide (MTT) assay. The MTT assay results showed that up to 200 μg/mL *E. hirta* was not cytotoxic to RAW 264.7 cells. However, the cell viability of these macrophages decreased to approximately 15% in all treated groups, including the ethanol-treated group (0 μg/mL *E. hirta* extract). This outcome may be due to the solvent (ethanol) itself because there was no difference in cell viability as the *E. hirta* concentration was increased from 25 to 200 μg/mL (Figure 6).

### 2.6. Anticancer Activity against an Acute Myeloid Leukemia Cell Line (HL-60) in Vitro

**Figure 7:**
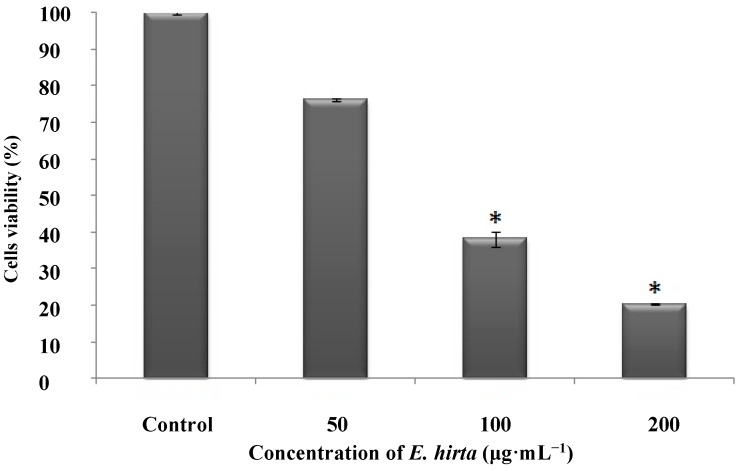
Anticancer activity of ethanol extract of *E. hirta* on myeloid leukemia cell line (HL-60) *in vitro*. Cells (2 × 10^4^) were incubated with *E. hirta* (50, 100, 200 μg/mL) at 37 °C for 24 h. Cells viabilities were evaluated using MTT assays. Data represent the means ± SDs of three independent experiments. * Significant reduction in cell viability at *p* < 0.05.

To investigate the anticancer properties of the *E. hirta* extract, HL-60 (4 × 10^4^ cells/mL in a 96 well plate) were incubated with *E. hirta* extract (50, 100, and 200 μg/mL) at 37 °C for 24 h, and cell viability was measured by the MTT assay. The results showed a significant dose-dependent increase in cancer cell death (Figure 7).

Many traditional medicinal plants in use are thought to prevent or reduce the progression of cancer. Medicinal plants contain certain compounds able to modify the physiological functions of cells and to act as anti-cancer drugs by arresting cancer cell proliferation. In the present study, *E. hirta* extract showed dose-dependent selective anticancer activity. Previous studies have also reported that *E. hirta* extract had selective cytotoxicity and efficacy against cancer cell lines such as Hep-2, malignant melanoma, and squamous cell carcinoma [37,38].

DDMP is reportedly a major contributor to anticancer effects against a colon cancer cell line [9]. Studies of DDMP revealed that it could modulate the activity of nuclear factor (NF)-κB and induce apoptosis of colon cancer cells [39,40]. Recent evidence has suggested that NF-κB activation is related to multiple aspects of oncogenesis [41]. NF-κB activity was found to be higher in colon cancer cell lines and in human tumor specimens [42]. DDMP reportedly suppressed anti-apoptotic genes (B-cell lymphoma [Bcl]-2) targeted by NF-κB and induced the expression of several pro-apoptotic genes ([Bcl]-2-associated X protein [Bax], cleaved caspase-3, and cleaved poly (ADP-ribose) polymerase [PARP]) [9]. Therefore, DDMP is a promising agent for the treatment of colon cancer.

In our study, DDMP constituted 2.54% of the area on the GC-MS analysis and may contribute to the anticancer activity of *E. hirta*. Although the exact anticancer mechanism of *E. hirta* is unclear, the present results suggested the need for further studies to isolate individual compounds from the crude extract and to study their effects on cancer signaling pathways.

## 3. Experimental Section

### 3.1. Plant Specimen and Preparation of Ethanolic Extract

Fresh whole *E. hirta* specimens including the leaves, flowers, stem, and root were harvested. The taxonomic identity of the plant was confirmed by the botanist of the Punjab Agriculture University, Ludhiana, India. The plants were washed in tap water and air-dried in the shade for 10 days. The sample was then dried in an oven at 60 °C for 1–2 days, ground to a fine powder using an electric blender, and stored in clean, labeled, airtight bottles. The powdered *E. hirta* (500 g) was extracted into 95% ethanol (500 mL) using a Soxhlet extractor for 1 h, and the extract was concentrated into a solid form using a rotary evaporator.

### 3.2. GC-MS Analysis

Chromatographic analysis was performed using a Shimadzu GC-MS (Model QP-2010, Shimadzu Co., Kyoto, Japan) in electron impact mode at a 70 eV ionization voltage, 230 °C injector temperature, and 280 °C detector temperature, using an Rtx-5MS capillary column (length, 30 m; internal diameter, 0.25 mm; and film thickness, 0.25 μm). The oven temperature was initially programmed at 80 °C (isothermal for 5 min), then increased to 200 °C at 5 °C/min and finally to 250 °C at 5 °C/min (isothermal for 16 min). Helium was used as the carrier gas at a flow rate of 1 mL/min and an injector volume of 1 μL, using a 1:10 split ratio. To isolate individual compounds from the plant specimen, the ethanolic extract was dissolved in ethanol (1 mg/mL), filtered through a 0.20-µm syringe filter (Advantec, Tokyo, Japan), and aliquots were injected onto the GC-MS. Mass spectra of compounds obtained from GC-MS were tentatively identified based on mass spectral data from the WILEY and NIST libraries. Further tentative identification was completed by comparing their mass spectra with those reported in previous publications.

### 3.3. DPPH Radical Scavenging Assay

DPPH radical scavenging activity was measured using an ESR spectrometer (JES-FA machine, JEOL, Tokyo, Japan) as described by Nanjo *et al.* [43]. Briefly, 60 μL of each extract sample was combined with 60 μL of DPPH (60 μmol/L) in ethanol. After 10 s of vigorous shaking, the solutions were transferred into 100-μL Teflon capillary tubes and fitted into the ESR spectrometer cavity. The spin adduct was determined on an ESR spectrometer exactly 2 min later under the following conditions: 3475 G central field, 100 kHz modulation frequency, 2 G modulation amplitude, 5 mW microwave power, 6.3 × 105 gain and 298 K temperature. Ethanol was used as a negative control and ascorbic acid was used as a positive control. The radical-scavenging activities were presented as percent (%) activities and calculated using the following equation:

Radical scavenging activity = [1 − (H1/H0)] × 100
(1)

where H1 and H0 are the relative peak heights of the radical signals of the samples and control, respectively. The IC_50_ (concentration providing 50% inhibition) was calculated graphically using a calibration curve in the linear range by plotting the extract concentration against the corresponding scavenging effect.

### 3.4. OH˙ Scavenging Assay

OH**˙** is generated via the Fenton reaction, and reacts rapidly with the nitrone spin trap 5,5-dimethyl-1-pyrroline N-oxide (DMPO); the resultant DMPO-OH adduct is detectable by ESR spectrometry [24]. Briefly, 0.2 mL each of 0.3 M DMPO, 10 mM FeSO_4_, and 10 mM H_2_O_2_ was added to the extract and control samples. The mixtures were then transferred into 100-μL Teflon capillary tubes. The spin adduct was measured on an ESR spectrometer exactly 2.5 min later under the following measurement conditions: 3475 G central field, 100 kHz modulation frequency, 2 G modulation amplitude, 1 mW microwave power, 6.3 × 105 gain, and a 298 K temperature. Ethanol was used as a negative control and ascorbic acid was used as a positive control.

### 3.5. On-Line HPLC-ABTS^+•^ Radical Scavenging Analysis

The ABTS^+•^ scavenging activity was determined using an HPLC system comprising a binary Waters 515 pump, Waters 2489 UV/vis and 2998 photodiode array detector, and a Waters 2707 auto sampler with an ABTS^+•^ radical analyzer interface (Waters, Milford, MA, USA). A 10-µL aliquot of the 5 mg/mL *E. hirta* sample was run at a 1 mL/min flow rate in an Atlantis T3 3 µM 3.0 × 150 mm column (Waters) using a gradient acetonitrile-water solvent system (0–40 min: 5:95 v/v, 40–50 min: 100% v/v, 50–60 min: 100% v/v), with absorbance detection at 254 nm. The ABTS^+•^ solution was prepared by diluting a 2 mM ABTS^+•^ stock solution containing 2.5 mM potassium persulfate 30-fold in HPLC-grade water. The solution was then incubated overnight in darkness at room temperature for radical stabilization. The separated analytes were sent to the “T” piece and reacted post-column with the ABTS^+•^ radical in a reaction coil at 40 °C. The reduction was detected as a negative peak by a UV detector at a wavelength of 734 nm.

### 3.6. Cell Culture

The murine macrophage cell line RAW 264.7 was purchased from the Korean Cell Line Bank (KCLB, Seoul, Korea) and cultured in Dulbecco’s modified Eagle’s medium (DMEM) supplemented with 100 U/mL penicillin, 100 μg/mL streptomycin, and 10% fetal bovine serum. The cells were incubated and maintained in a 5% CO_2_ atmosphere at 37 °C. The cells were sub-cultured every 2 days, and exponential phase cells were used throughout the experiments.

### 3.7. Determination of NO Production

RAW 264.7 cells (1 × 10^5^ cell/mL) were placed in a 24-well plate for 24 h before pre-incubation with various concentrations of *E. hirta* extract (25, 50, 100 and 200 µg/mL) at 37 °C for 1 h. The cells were incubated at the same temperature for an additional 24 h with 1 μg/mL LPS produced by Escherichia coli 0111:B4. After the incubation, the quantity of nitrite in the culture medium was measured as an indicator of NO production [44]. Briefly, 100 μL of the cell culture medium was mixed with 100 μL of Griess reagent (1% sulfanilamide and 0.1% naphthylethylenediamine dihydrochloride in 2.5% phosphoric acid), the mixture was incubated at room temperature for 10 min, and the optical density at 540 nm was measured using a microplate reader (Model-680, Bio-Rad, Hercules, CA, USA). LPS-incubated cells and cells without *E. hirta* treatment were used as controls. Fresh culture medium was used as a blank sample in every experiment.

### 3.8. Cytotoxicity Assessment by MTT Assay

The cytotoxicity of the plant extract towards RAW 264.7 cells was determined using a colorimetric MTT assay. Cells were seeded into a 24-well plate at 1 × 10^5^ cells/mL and 24 h later, they were treated with various concentrations of *E. hirta* extract (50, 100 and 200 µg/mL). The cells were then incubated for an additional 24 h at 37 °C. MTT stock solution (50 µL; 2 mg/mL in phosphate-buffered saline, PBS) was added to each well to provide a total reaction volume of 250 µL, incubated for 3 h, and the supernatants were aspirated. The formazan crystals in each well were dissolved in 200 µL dimethylsulfoxide (DMSO), and the absorbance was measured in a microplate reader (Model-680, Bio-Rad) at 540 nm. Cells not treated with *E. hirta* extract served as the control. Fresh culture medium was used as a blank sample in every experiment.

### 3.9. Anticancer Activity Assay

The cell growth inhibitory activity (cytotoxicity) of the extract at different concentrations was assessed in HL-60 cancer cells by MTT assay. Suspended promyeloid (HL-60) cells were seeded into a 96-well plate at 2 × 10^4^ cells/mL along with various concentrations of *E. hirta* extract (50, 100, and 200 µg/mL) and incubated for 24 h before MTT treatment. MTT stock solution (50 µL; 2 mg/mL in PBS) was added to each well to produce a total reaction volume of 250 µL. After 4 h of incubation, the plates were centrifuged for 10 min at 2000 rpm, the supernatants were aspirated, and the formazan crystals in each well were dissolved in DMSO. The amount of purple formazan was determined by measuring the absorbance at 540 nm. *E. hirta* was not added to the control samples.

### 3.10. Statistical Analysis

Analytical data are reported as the mean ± standard deviation (SD) of triplicate independent measurements. The statistical significance level was set at 5% (*p <* 0.05).

## 4. Conclusions

The current study indicated that *E. hirta* ethanol extract had antioxidant, anti-inflammatory, and anticancer activities. The present findings of *E. hirta*-mediated inhibition of NO production may be of clinical significance for host defense against infections and for the treatment of inflammatory diseases, cancer, and ageing. Future studies are needed to isolate the individual chemical fractions of *E. hirta*, evaluate their molecular mechanisms of action, and classify the fractions according to their antioxidant, anti-inflammatory, and anticancer activities, potentially providing novel means to regulate cellular activity using this herb. The current results suggested that the *E. hirta* ethanol extract had potent antioxidant effects that may contribute to its anti-inflammatory and anticancer properties. Overall, the many potential clinical applications of *E. hirta* provide a strong rationale for further investigation of the molecular mechanisms underlying the biological effects of this plant.
